# Supplementary material for: Reporting quality in preclinical animal experimental research in 2009 and 2018: A nationwide systematic investigation
Source: PLoS One. 2022 Nov 3;17(11):e0275962. doi: 10.1371/journal.pone.0275962 (PMC9632797; doi:10.1371/journal.pone.0275962)
Supplement: S2 File — (DOCX) [file pone.0275962.s002.docx]

**Supplementary material**

**Exclusions based on title and abstract and full text screening**

Manuscripts containing or characterized by farm or wild animal target, environmental investigations, human studies, not in vivo, not primary paper (review, abstract, proceedings, supplement, image, PhD thesis etc.), no intervention, in vitro, invertebrate, no abstract, exploratory studies without hypothesis testing statistical analyses and interventions, full text not available or no author affiliation were excluded in the pre-screening selection phase or the full-text screening phase as shown in the table below.

The number of publications excluded for each criteria following eligibility screening:
