# Supplementary material for: Reporting quality in preclinical animal experimental research in 2009 and 2018: A nationwide systematic investigation
Source: PLoS One. 2022 Nov 3;17(11):e0275962. doi: 10.1371/journal.pone.0275962 (PMC9632797; doi:10.1371/journal.pone.0275962)
Supplement: S1 Table — (DOCX) [file pone.0275962.s005.docx]

**Table 1**

**Data extraction form: Operationalized items to facilitate assessment of reporting quality.**

The form consists of 10 items with questions to facilitate assessment of reporting. Each item has only one answer option, which could be “Yes” or “No”. Items are scored “Yes” if the item is reported or “No” when there is no reporting of the item or when criteria for “Yes” are not met (See S2 Table for criteria for “Yes” for each item). In case of difficult scoring decisions (e.g. the item is partially reported, or items are absent but reported (e.g., authors reported randomization was not conducted)) the study is scored as “Yes” and notes are saved to provide clarification about the items. Thereafter, the reporting quality is discriminated by a numerical score. A score of 3, 2, 1, or 0 is given according to the quality of information pertaining to each question. For example, for the information regarding whether experiments were randomized, a score of 3, 2, 1, 0 corresponds to reported performed and method disclosed, reported performed, reported ‘not performed’ (statement), and not reported or criteria for reported not met, respectively.

| **Item** | **Question** | **Score** | | | |
| --- | --- | --- | --- | --- | --- |
|  |  | **“No” (Not reported)** | **“Yes” (Reported)** | | |
|  |  | **0** | **1** | **2** | **3** |
| Sample size  (other bias) | Do authors report an exact sample size? | Not reported | Reported | - | - |
| Sample size calculation  (other bias) | Do authors describe how the sample size was chosen? | Not reported | Reported ‘not performed’ | Reported performed | Reported performed and calculation disclosed |
| Randomization  (selection bias) | Do authors include a statement of randomization? | Not reported | Reported ‘not performed’ | Reported performed | Reported performed and method disclosed |
| Blinded experiment conduction (performance bias) | Do author include a statement of whether caretakers were blinded? | Not reported | Reported ‘not performed’ | Reported performed | - |
| Blinded outcome assessment  (detection bias) | Do authors include a statement of whether experimenters were blinded? | Not reported | Reported ‘not performed’ | Reported performed | - |
| Attrition I  (attrition bias) | Is the number of samples or animals at the end of the study stated? | Not reported | Reported but not for all analyses | Reported with exact numbers for all analyses | - |
| Attrition II  (attrition bias) | Is the number of samples or animals at the end of the study the same as the sample size at the beginning of the study? | Not reported | Reported | - | - |
| Exclusion  (attrition bias) | Is there a clear description of missing or excluded samples or animals from the analysis? | Not reported | Reported but without numbers and reason | Reported ‘no exclusions’ or ‘all included’ | Reported with number and reason for exclusion for all analyses |
| Health status  (other bias) | Is the health status of the animals at the beginning of the study stated? | Not reported | Reported without further information | Reported and detailed information disclosed | - |
| Conflicts of interest  (other bias) | Is there a declaration of conflicts of interest? | Not reported | Reported and conflict of interest present | Reported and conflict of interest absent | - |
