# Supplementary material for: Reporting quality in preclinical animal experimental research in 2009 and 2018: A nationwide systematic investigation
Source: PLoS One. 2022 Nov 3;17(11):e0275962. doi: 10.1371/journal.pone.0275962 (PMC9632797; doi:10.1371/journal.pone.0275962)
Supplement: S2 Table — (DOCX) [file pone.0275962.s006.docx]

**Table 2**

**Criteria for the assessment of the reporting quality^[[1]](#footnote-1)^**

**Sample size**

| Criteria for a judgement of “Yes” (Reported) | An exact sample size is given at the beginning of the study before group assignment.  The sample size is given as an exact number (not as a range), total or for each experimental group/condition | Quote: *“A total of 40 female pigs were included.”* |
| --- | --- | --- |

**Sample size calculation**

| Criteria for a judgement of “Yes” (Reported) | Reported ‘not performed’ | A statement that no calculation was performed | Quote: *“No statistical methods were used to pre-determine sample sizes.”* |
| --- | --- | --- | --- |
|  | Reported performed | Reported performed but no calculation method is disclosed, or the sample size was chosen based on experience | Quote: *“The group sizes were chosen based on previous studies and experience.”* |
|  | Reported performed and calculation disclosed | Reporting power, effect size and/or software used | Quote: *“power analysis was performed to determine the appropriate sample size using the online BioMath software (*[*http://www.biomath.info/).*](http://www.biomath.info/)) *The effective sample size for these experiments was determined to be 6 per experimental group.”* |

**Randomization**

| Criteria for a judgement of “Yes” (Reported) | Reported ‘not performed’ | It is stated that randomization is not performed | None |
| --- | --- | --- | --- |
|  | Reported performed | A statement about randomization is reported | Quote: *“animals were randomly allocated.”* |
|  | Reported performed and method disclosed | A description of the method of randomization with which samples or animals are allocated to experimental groups is reported | Quote: *“Computer-generated random numbers were used with a unique code linking to the individual animal.”* |

**Blinded experiment conduction**

| Criteria for a judgement of “Yes” (Reported) | Reported ‘not performed’ | It is stated that blinding is not performed | Quote: *“The administration of treatments was not blinded.”* |
| --- | --- | --- | --- |
|  | Reported performed | Blinding is reported to be performed | Quote: *“An independent study assistant… prepared visibly identical syringes...”* |

**Blinded outcome assessment**

| Criteria for a judgement of “Yes” (Reported) | Reported ‘not performed’ | It is stated that blinding is not performed | Quote: *“Researchers were not blinded to the treatment groups.”* |
| --- | --- | --- | --- |
|  | Reported performed | Blinding is reported to be performed | Quote: *“The experimenter was blinded to the treatment the animals had received.”* |

**Attrition I**

| Criteria for a judgement of “Yes” (Reported) | Reported but not for all analyses | The number of samples or animals at the end of the study are not reported for all analyses or given as ranges | Quote: *“7-8 per treatment group.”* |
| --- | --- | --- | --- |
|  | Reported with exact numbers for all analyses | The number of samples or animals at the end of the study are reported for all analyses with exact numbers (no ranges) | Quote: *“All animals completed the study…assuring n = 8 for all investigated groups.”* |

**Attrition II**

| Criteria for a judgement of “Yes” (Reported) | Numbers of samples or animals are reported to be the same at the beginning and end of the study. | Comment: *“n=8 in beginning, n=8 for all analyses (figures).”* |
| --- | --- | --- |

**Exclusions**

| Criteria for a judgement of “Yes” (Reported) | Reported but without numbers and reason for exclusion | Exclusions of samples or animals are reported but no exact numbers or reasons for exclusion are given | Quote: *“Due to a few cases of unexpected death, animal numbers of groups were in some instances reduced to n = 9 and once to n = 8.”* |
| --- | --- | --- | --- |
|  | Reported no exclusions/all included | A statement of no exclusions of animals or all included in the analyses is reported | Quote: “*No mice were excluded from this study.”* |
|  | Reported with numbers and reason for exclusion for all analyses | Exclusions of samples or animals are reported with exact numbers and reason for exclusion for all analyses | Quote: *“All the pigs tolerated the surgical procedure, except for one who had an incisional hernia postoperatively and was immediately euthanized.*  *Consequently, seven pigs were observed for 6 weeks, and eight pigs were observed for 12 weeks before termination.”* |

**Health status**

| Criteria for a judgement of “Yes” (Reported) | Reported without further information | The health status of the animals is stated at the beginning of the study (*e.g. the animals were housed in a SPF facility, the animals appeared healthy upon clinical examination etc.)* | Quote: *“..52 young adult virgin female Wistar rats…were maintained and monitored in a specific pathogen-free environment.”* |
| --- | --- | --- | --- |
|  | Reported and detailed information disclosed | Detailed information of the health status of the animals is provided (e.g. a health report) | Quote: *“The facility followed the Federation for European Laboratory Animal Science Associations (FELASA) guidelines for health monitoring in rodent facilities, 15 where sentinel mice had tested positive for Helicobacter spp. but none of the other pathogens on the FELASA list.”* |

**Conflict of interest**

| Criteria for a judgement of “Yes” (Reported) | Reported and conflict of interest present |  | Quote: *“The authors disclose the following: P.W. and S.E. are members of the Project Team at the Institute of Cancer Research who developed AUY922 in collaboration with Vernalis. All other authors disclose no conﬂicts”* |
| --- | --- | --- | --- |
|  | Reported and conflict of interest absent |  | Quote: *“The authors declare that the research was conducted in the absence of any commercial or ﬁnancial relationships that could*  *be construed as a potential conﬂict of interest.”* |

1. Modified from Cochrane Handbook for Systematic Reviews of Interventions, version 5.2.0 (updated June 2017), Cochrane, 2017 [↑](#footnote-ref-1)
