# Supplementary material for: Reporting quality in preclinical animal experimental research in 2009 and 2018: A nationwide systematic investigation
Source: PLoS One. 2022 Nov 3;17(11):e0275962. doi: 10.1371/journal.pone.0275962 (PMC9632797; doi:10.1371/journal.pone.0275962)
Supplement: Attachment — Submitted filename: Response to Reviewers.docx [file pone.0275962.s008.docx]

**Response to reviewers:**

Dear Academic Editor, Dr. Gilllian Currie

Thank you for your continued interest in our paper (PONE-D-21-20844R1) and the effort that you have put into reviewing the revised manuscript. Please find our point-by-point reply below. In addition to this rebuttal letter labeled 'Response to Reviewers', we have uploaded a marked-up copy of the manuscript that highlights changes made to the original version labeled 'Revised Manuscript with Track Changes', and an unmarked version of the revised paper without tracked changes labeled 'Manuscript' as requested.

**Response to Dr Currie’s comments and requests:**

Thank you for your efforts revising your manuscript in response to our suggestions. My main concern is that your data should be made available- this is a requirement for publication. PLOS journals require authors to make all data necessary to replicate their study’s findings publicly available without restriction at the time of publication. The specific details can be found here: https://journals.plos.org/plosone/s/data-availability#loc-unacceptabledata-access-restrictions

Response:

Thank you for responding to our request. Time has passed and at this time point we are fully able to make all data necessary to replicate our study’s findings available. So it is no longer an issue for us to keep the data restricted. The datasets are currently stored in The Open Science Framework repository <https://osf.io> and thus ready to be made publicly available upon PLOS One’s request. Additionally, the Supporting information file, S3 File is renamed “S3 File: Agreement data” in line 501 and now only contains our agreement data.

I do not believe that a PRISMA checklist is necessary for this study but it is useful to include the graphical representation of the flow of studies (Figure 1).

Response:

Thank you for agreeing on this matter. We omitted the PRISMA checklist from the supporting information and maintained the graphical presentation of the flow of studies (Figure 1) in the manuscript and submitted the manuscript. We were then notified to include the checklist in order for the submission process to proceed and swiftly responded your decision in the “Enter comments” prior to re-submitting our manuscript. However, omitting the checklist resulted in a new “revisions sent back to author” notification with a request for the checklist. Thus, to resolve this and speed up the review process (currently > 1 year), we have included the PRISMA checklist and stamped it with “Not to be included in the manuscript”.

1. Line 72-78: “Previous research has investigated the prevalence of reporting of measures to reduce the risk of bias for specific animal disease models or subjects of interest [29-32]. Other previous evaluations of preclinical reporting have provided an overview of the reporting status of items related to the internal validity or rigor of these experiments (e.g. blinding and randomization) [33]. Taking a step further, this study investigates the reported information's level of detail by assessing preclinical studies within all animal experimental research fields with one or more authors affiliated with Danish research institutions.” I suggest that you remove the “Taking a step further” and instead “This study investigates the reported information's level of detail by assessing preclinical studies within all animal experimental research fields with one or more authors affiliated with Danish research institutions.”.

Response:

Thank you for this comment. The sentence is corrected according to your request. You will find the sentence: “This study investigates the reported information's level of detail by assessing preclinical studies within all animal experimental research fields with one or more authors affiliated with Danish research institutions.” in line 75-78.

2. Line 49-52:

I do not think that the sentence “Internal validity refers to how appropriate methodologies safeguarding against systematic errors (bias) are implemented in the design, conduct, and analysis of an experiment” is accurate (this is not a definition of internal validity that I am familiar with). Amend to clarify e.g. define internal validity and then state that methodologies to safeguard against bias can be implemented to increase internal validity.

Response:

We agree that the sentences indeed need an additional revision in order to be clear and concise and thank you for underscoring this fact. We suggest the following:

Line 43-46: “A prevalent issue is unsatisfactory internal validity. Internal validity is the extent to which a design and conduct of a study eliminates the possibility of systematic errors (bias) [4]. Appropriate methodologies safeguarding against systematic errors can be implemented in the design, conduct, and analysis of an experiment in order to increase the internal validity [4].”

3. Line 74-75: “In this context, realization is necessary already at the planning stages to guarantee good reporting in the end.” Amend sentence to clarify meaning. Response: Thank you for this comment. We have clarified the meaning by rewriting the sentence: Line 68-71: “The implementation may be hindered by the lack of engagement of multiple stakeholders who all must engage in improving the reporting quality. In this context, researchers’ use of the guideline is necessary already in the planning stages to guarantee good reporting.”. I suggest that the wording is changed to clarify meaning. If I have understood your meaning correctly then I suggest the wording “In this context, the use of the ARRIVE guidelines by researchers is necessary at the planning stage to help improve experimental design and, in turn, improve reporting.”

Response:

Thank you for this comment. We have clarified the meaning of the sentence by revising according to your suggestion: “The implementation may be hindered by the lack of engagement of multiple stakeholders who all must engage in improving the reporting quality. In this context, the use of the ARRIVE guideline by researchers is necessary already at the planning stage to help improve experimental design and, in turn, improve reporting.” in line 67-71.

4. Line 122: Please give details on how you defined and identified an exploratory study. Response: We defined an exploratory study as research connecting ideas to understand cause-effect and investigating novel relevant questions that have not previously been thoroughly studied. These studies do not test but generate hypotheses. To clarify this we have added “…(i.e. studies investigating novel questions and hypothesis-generating studies)…”. The sentence now states: Line 119-123: “The exclusion of studies was based on the following exclusion criteria: science related to farming, wild animals or invertebrates, environment, human (clinical) studies, in vitro research, not primary papers/publications, lack of abstract or full text, exploratory studies (i.e. studies investigating novel questions and hypothesis-generating studies), and studies containing no intervention or no Danish author affiliation.” I am still not clear as to how an exploratory study was identified. How did you decide it was a novel question? Or hypothesis-generating? Did the authors have to state this in their manuscript?

Response:

Thank you for your comment. Exploratory studies were identified through study author statements that the study was explorative, or the study was assessed to be hypothesis-generating and performed to e. g. investigate novel questions, develop models or investigate and validate new animal models, look for biomarkers, explore pharmacokinetics/biodistribution, gene identification/expression, or mapping of receptors or microcirculation”.

To clarify, we have amended the sentence in line 119-125: “The exclusion of studies was based on the following exclusion criteria: science related to farming, wild animals or invertebrates, environment, human (clinical) studies, in vitro research, not primary papers/publications, lack of abstract or full text, studies containing no intervention or no Danish author affiliation, and exploratory studies (the latter studies were identified through study author statements that the study was explorative, or studies were assessed to investigate novel questions and to be hypothesis-generating).”

We are looking forward to your reply.

On behalf of the authors.

Kind regards,

Dr. Kousholt
